# Bias-corrected CMIP6 global dataset for dynamical downscaling of the historical and future climate (1979–2100)

**DOI:** 10.1038/s41597-021-01079-3

**Published:** 2021-11-04

**Authors:** Zhongfeng Xu, Ying Han, Chi-Yung Tam, Zong-Liang Yang, Congbin Fu

**Affiliations:** 1 RCE-TEA, Institute of Atmospheric Physics, Chinese Academy of Sciences, Beijing, 100029 China; 2 Earth System Science Programme, The Chinse University of Hong Kong, Hong Kong, China; 3 Department of Geological Sciences, Jackson School of Geosciences, The University of Texas at Austin, Austin, Texas USA; 4 School of Atmospheric Sciences, Nanjing University, 210093 Nanjing, China

**Keywords:** Hydrology, Environmental impact, Projection and prediction

## Abstract

Dynamical downscaling is an important approach to obtaining fine-scale weather and climate information. However, dynamical downscaling simulations are often degraded by biases in the large-scale forcing itself. We constructed a bias-corrected global dataset based on 18 models from the Coupled Model Intercomparison Project Phase 6 (CMIP6) and the European Centre for Medium-Range Weather Forecasts Reanalysis 5 (ERA5) dataset. The bias-corrected data have an ERA5-based mean climate and interannual variance, but with a non-linear trend from the ensemble mean of the 18 CMIP6 models. The dataset spans the historical time period 1979–2014 and future scenarios (SSP245 and SSP585) for 2015–2100 with a horizontal grid spacing of (1.25° × 1.25°) at six-hourly intervals. Our evaluation suggests that the bias-corrected data are of better quality than the individual CMIP6 models in terms of the climatological mean, interannual variance and extreme events. This dataset will be useful for dynamical downscaling projections of the Earth’s future climate, atmospheric environment, hydrology, agriculture, wind power, etc.

## Abstract (summary)

**Table:** 

Measurement(s)	temperature of air • atmospheric wind • humidity • geopotential height • temperature of sea surface • surface pressure • sea level pressure
Technology Type(s)	climate model • meteorological reanalysis
Factor Type(s)	zonal wind • meridional wind • temporal interval • geographic location
Sample Characteristic - Environment	climate system • climate change
Sample Characteristic - Location	global

Machine-accessible metadata file describing the reported data: 10.6084/m9.figshare.16802326

## Background & Summary

Projections of the Earth’s future climate at a finer scale are of great importance in climate-related studies, such as studies of climate extremes, water resources, agriculture, air quality and wind power. Common approaches to producing high-resolution projection data include interpolation^1,2^, statistical downscaling^3,4^, dynamical downscaling^5–8^ and hybrid statistical–dynamical downscaling^9,10^. Unlike interpolation and statistical downscaling, dynamical downscaling can represent various physical processes and their interactions in the climate system and can generate a full set of dynamically consistent high-resolution climate data. Traditional dynamical downscaling of the future climate involves integrating a regional climate model (RCM) with the initial and lateral boundary conditions from a general circulation model (GCM)^6,11^. This traditional dynamical downscaling approach has been widely reported^12,13^. However, GCMs are known to have significant biases, which propagate into RCMs through the lateral boundary and thus degrade the downscaled simulations^14–16^.

In recent years, GCM bias corrections have recently become an important topic in dynamical downscaling studies and many GCM bias correction methods have been developed, including GCM mean bias correction^17–19^, GCM mean and variance bias corrections^16,20^, trend-preserving bias correction^21,22^, quantile-quantile correction^23^, nested bias correction^24^ and multi-model ensemble (MME) mean-based bias correction^25^. Compared with the traditional dynamical downscaling approach, these bias correction methods significantly improve downscaling simulations. For example, GCM mean bias correction clearly improved the dynamical downscaling simulation of tropical cyclones over the North Atlantic Ocean^18,19^. The introduction of variance bias correction further improved the downscaled interannual variability and extreme events^16^. MME mean-based bias correction is expected to generate a more credible downscaling projection of the Earth’s future climate^25^.

However, the current GCM bias correction methods still have limitations^26,27^. For example, Bruyère *et al*.^19^ only corrected the GCM mean bias and left the other biases untouched. In terms of long-term dynamical downscaling, variance bias correction may inappropriately modify the GCM trend^16,21^. Quantile–quantile correction cannot retain the intervariable dependencies and introduces an additional bias in the spatial gradient of variables^23,28^. Note that most bias correction methods are applied to a single model dataset, which suffers greater uncertainty in terms of the projection of the Earth’s future climate. Dai *et al*.^25^ applied GCM bias correction to the MME mean of the Coupled Model Intercomparison Project Phase 5 (CMIP5) models, but only corrected the GCM mean bias.

It is well known that the climate projections generated by GCMs are still highly uncertain, especially for precipitation and its extremes^29^. The sources of uncertainties include the future emission scenarios, internal climate variability and model uncertainty^30^. These uncertainties hinder the application of climate projections in impact studies, such as the impact of future climate change on water resources and agriculture. We constructed a bias-corrected GCM dataset based on a novel GCM bias correction method. Our method takes advantage of the non-linear trend of the ensemble mean of 18 Coupled Model Intercomparison Project Phase 6 (CMIP6) models to give a more reliable projection of the long-term climate trend. In addition, we also correct the GCM climatological mean and interannual variance biases based on the European Centre for Medium-Range Weather Forecasts Reanalysis 5 (ERA5) dataset. The bias corrections were applied to historical simulations over the time period 1979–2014 and two future scenarios of SSP245 and SSP585 over the time period 2015–2100. This bias-corrected dataset provides high-quality large-scale forcing for dynamical downscaling simulations and will improve the reliability of future projections of the regional climate and environment.

## Methods

### Data acquisition

We used the monthly data derived from the CMIP6 historical experiment and future scenarios of SSP245 and SSP585^31^. Our bias correction method decomposes GCM and reanalysis data into long-term trends and anomalies. The long-term trend is computed using the MME mean derived from 18 CMIP6 models over historical and future time periods (Table 1). The use of MME is expected to significantly reduce the uncertainty in projections of the future climate compared with individual GCMs. However, the MME mean largely cancels out the internal climate variability. To retain the internal climate variability, the anomalies are computed using one of the CMIP6 models. Previous studies have suggested that the MPI-M Earth system models show a generally good performance in the simulation of the sea surface temperature (SST) and atmospheric circulation among the CMIP5 and CMIP6 models^32–34^. We therefore selected the six-hourly dataset produced by the high-resolution version of the MPI-M Earth system model (MPI-ESM1-2-HR) in CMIP6 to generate the weather and interannual variability of the large-scale forcing data. The MPI-ESM1-2-HR is configured with a horizontal grid spacing of 100 km in the atmosphere and 40 km in the ocean^35^. Compared with its low-resolution counterpart MPI-ESM1.2-LR, MPI-ESM1-2-HR shows an improved performance in mid-latitude storm track dynamics, atmospheric blocking, and quasi-biennial oscillation, although the improvement in the mean state is relatively modest^35–37^. The variables used include the upper air temperature, zonal wind, meridional wind, relative humidity, geopotential height, as well as the surface pressure, sea-level pressure and SST.

**Table 1:** CMIP6 models used in this study.

No.	Model	Institution	Approximate grid spacing
1	ACCESS-CM2	Commonwealth Scientific and Industrial Research Organisation (Australia)	1.875° × 1.25°
2	ACCESS-ESM1–5	Commonwealth Scientific and Industrial Research Organisation (Australia)	1.875° × 1.25°
3	CanESM5	Canadian Centre for Climate Modelling and Analysis (Canada)	2.81° × 2.81°
4	BCC-CSM2-MR	Beijing Climate Center (China)	1.125° × 1.125°
5	FGOALS-f3-L	Institute of Atmospheric Physics, Chinese Academy of Sciences (China)	1.25° × 1°
6	FGOALS-g3	Institute of Atmospheric Physics, Chinese Academy of Sciences (China)	2° × 2.25°
7	EC-Earth3	European EC-Earth Consortium (Europe)	0.70° × 0.70°
8	EC-Earth3-Veg	European EC-Earth Consortium (Europe)	0.70° × 0.70°
9	IPSL-CM6A-LR	Institute Pierre Simon Laplace (France)	2.5° × 1.26°
10	AWI-CM-1-1-MR	Alfred Wegener Institute, Helmholtz Centre for Polar and Marine Research (Germany)	0.94° × 0.94°
11	MPI-ESM1-2-HR	Max Planck Institute for Meteorology (Germany)	0.94° × 0.94°
12	MPI-ESM1-2-LR	Max Planck Institute for Meteorology (Germany)	1.875° × 1.875°
13	MIROC6	Japan Agency for Marine-Earth Science and Technology (Japan)	1.41° × 1.41°
14	MRI-ESM2-0	Meteorological Research Institute, Japan Meteorological Agency (Japan)	1.125° × 1.125°
15	NorESM2-LM	Norwegian Climate Center (Norway)	2.5° × 1.875°
16	CESM2	Climate and Global Dynamics Laboratory, National Center for Atmospheric Research (USA)	1.25° × 0.94°
17	CESM2-WACCM	Climate and Global Dynamics Laboratory, National Center for Atmospheric Research (USA)	1.25° × 0.94°
18	GFDL-ESM4	Geophysical Fluid Dynamics Laboratory, National Oceanic and Atmosphere Administration (USA)	1.25° × 1.0°

We also used the European Centre for Medium-Range Weather Forecasts ERA5 reanalysis dataset from 1979 to 2014, which is produced using 4D-var data assimilation^38^. The ERA5 dataset has a finer horizontal grid spacing of 31 km and more assimilated observations than the ERA-Interim dataset. The ERA5 dataset shows a considerable improvement in the temperature, wind, and humidity in the troposphere, but not in the stratosphere, compared with the ERA-Interim dataset. Both the ERA5 and CMIP6 datasets were re-gridded to a horizontal grid spacing of (1.25° × 1.25°) using bilinear interpolation.

### GCM bias-correction method

**Fig. 1:**
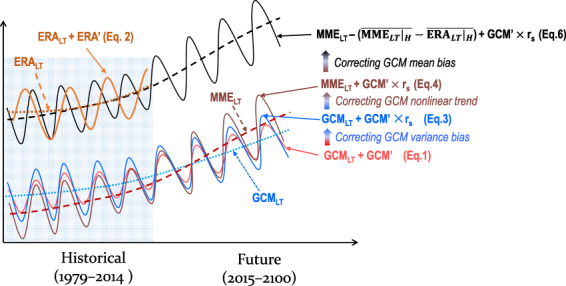
Schematic chart of the process of GCM bias corrections. The abbreviations are as given in the text for Eqs. 1–6. The orange curve represents the ERA5 data. The dashed and dotted lines denote the non-linear trends derived from the MME and the MPI-ESM1-2-HR model (ERA5 dataset), respectively. The red curve indicates the original GCM data. The blue curve represents the GCM data after variance bias correction. The brown curve was obtained by replacing the non-linear trend of the blue curve with that derived from the MME. The black curve is the final bias-corrected GCM data.

The bias corrections were applied to the six-hourly data. Each non-leap year contains 1460 data points. For each six-hour period and day of the year, the ERA reanalysis (Fig. 1, orange curve) and GCM (Fig. 1, red curve) outputs can be broken down into a long-term non-linear trend plus an interannual perturbation term:

1 $${\rm{GCM}}={{\rm{GCM}}}_{LT}+{\rm{GCM}}{\prime} $$

2 $${\rm{ERA}}={{\rm{ERA}}}_{LT}+ERA{\prime} $$

The non-linear trend was calculated using the ensemble empirical model decomposition (EEMD) method^39^. Unlike the decomposition method used in previous studies, which broke the GCM into a climatological mean or linear trend plus a perturbation term^16,17,21^, our decomposition method excludes the long-term non-linear trend in the perturbation term, which can avoid inappropriate modification of the long-term trend during the variance bias correction. Note that the GCM may also overestimate or underestimate the amplitude of interannual variations. This bias can be measured by the ratio of the GCM variance to the reanalysis variance. We assume that the variance bias remains the same from the historical period to a future period. Thus, we can correct the variance bias by multiplying the perturbation term by a scaling factor, r_*s*_:

3 $${{\rm{GCM}}}_{v}={{\rm{GCM}}}_{LT}+{\rm{GCM}}{\prime} \times {{\rm{r}}}_{s}$$

where ${{\rm{r}}}_{s}={\sigma }_{ERA}/{\sigma }_{GCM}$ is the ratio of the standard deviation of the detrended reanalysis data to that of the detrended GCM data over the historical time period. As the standard deviations are computed using the detrended data, we adjusted the variance of the interannual and interdecadal variations so that the non-linear trend remained unchanged (Fig. 1, blue curve). This is because the long-term trend may be changed inappropriately if we use the standard deviation of the original time series to adjust the variance^21^.

It is known that single-GCM projections of the long-term trend give greater uncertainty than the MME mean. To reduce the uncertainty of future projections, we replaced the long-term non-linear trend derived from the single GCM with that derived from the MME in Eq. (3). The new GCM data (Fig. 1, brown curve) can therefore be rewritten as:

4 $${{\rm{GCM}}}_{vt}={{\rm{MME}}}_{LT}+{\rm{GCM}}{\prime} \times {{\rm{r}}}_{s}$$

where MME_*LT*_ is the non-linear trend of the MME computed with the EEMD over the historical–future time period. Equation (4) can be further rearranged according to:

5 $${{\rm{G}}{\rm{C}}{\rm{M}}}_{vt}={{\rm{M}}{\rm{M}}{\rm{E}}}_{LT}+(\bar{{{\rm{M}}{\rm{M}}{\rm{E}}}_{LT}{|}_{H}}-\bar{{{\rm{E}}{\rm{R}}{\rm{A}}}_{LT}{|}_{H}})-(\bar{{{\rm{M}}{\rm{M}}{\rm{E}}}_{LT}{|}_{H}}-\bar{{{\rm{E}}{\rm{R}}{\rm{A}}}_{LT}{|}_{H}})+{\rm{G}}{\rm{C}}{\rm{M}}{\rm{{\prime} }}\times {{\rm{r}}}_{s}$$

The subscript *H* represents the historical time period (1979–2014), whereas the overbar indicates the climatological mean. $(\bar{{{\rm{M}}{\rm{M}}{\rm{E}}}_{LT}{|}_{H}}-\bar{{{\rm{E}}{\rm{R}}{\rm{A}}}_{LT}{|}_{H}})$ is the mean bias of the long-term trend of the GCM data relative to that of the reanalysis dataset over the historical time period, which can be removed to correct the GCM mean bias. Further bias-corrected data can be constructed as follows:

6 $$\begin{array}{ccc}{{\rm{G}}{\rm{C}}{\rm{M}}}_{mvt} & = & {{\rm{M}}{\rm{M}}{\rm{E}}}_{LT}-(\bar{{{\rm{M}}{\rm{M}}{\rm{E}}}_{LT}{|}_{H}}-\bar{{{\rm{E}}{\rm{R}}{\rm{A}}}_{LT}{|}_{H}})+{\rm{G}}{\rm{C}}{\rm{M}}{\rm{{\prime} }}\times {{\rm{r}}}_{s}\\  & = & \bar{{{\rm{E}}{\rm{R}}{\rm{A}}}_{LT}{|}_{H}}+({{\rm{M}}{\rm{M}}{\rm{E}}}_{LT}-\bar{{{\rm{M}}{\rm{M}}{\rm{E}}}_{LT}{|}_{H}})+{\rm{G}}{\rm{C}}{\rm{M}}{\rm{{\prime} }}\times {{\rm{r}}}_{s}\end{array}$$

The bias-corrected six-hourly GCM data over the future period therefore have a base climate provided by the reanalysis dataset over the historical period, with the change in future climate relative to the historical climatology generated by the MME and the future bias-corrected weather and climate variability derived from a single GCM (Fig. 1, black curve). Equation (6) is the final equation that corrects the GCM mean and variance biases after replacing the single GCM non-linear trend with the MME non-linear trend (hereafter MVT bias correction method).

The EEMD method used to compute the non-linear trend is very time consuming if we apply it to process global six-hourly 3D datasets over multiple decades. To save computing time, we assumed that the long-term non-linear trends remained the same for each six-hourly/daily value of variables within the same month. The climatological mean of the detrended data, $\frac{1}{N}{\sum }_{i=1}^{N}\left(GCM-GC{M}_{LT}\right)$, is therefore not exactly equal to zero because *GCM* and *GCM*_*LT*_ are derived from six-hourly data and monthly data, respectively. Before correcting the variance biases, we computed the anomaly of the detrended GCM data at each six-hourly interval/day of the year:

7 $${\rm{GCM}}{\prime} =\left(GCM-GC{M}_{LT}\right)-\frac{1}{N}{\sum }_{i=1}^{N}\left(GCM-GC{M}_{LT}\right)$$

The first term on the right-hand side of Eq. (7) represents the detrended GCM data. The standard deviation of each six-hour interval and day of the year was calculated across 36 years from 1979 to 2014. Note that extreme events (e.g. tropical cyclones) can strongly affect the interannual standard deviation of many variables, such as the sea-level pressure, geopotential height and wind. As tropical cyclones occur in different locations in the CMIP6 models and the reanalysis dataset, the ratio of the standard deviation in the grid cells with tropical cyclones is unrealistically different from its surrounding regions. To remove the unrealistic ratios of the standard deviation, we first calculated the original standard deviation with all 36 years of data and then recalculated the standard deviation after removing the years with anomalies greater than three times the original standard deviation.

In addition to the surface and upper air variables, an RCM also requires the moisture and temperature in different soil layers as the initial conditions. Similar to the lateral boundary condition, the initial soil conditions were constructed with the ensemble mean of 15 CMIP6 models. As a result of their incomplete soil moisture data, the AWI-CM-1-1-MR, FOGALS-f3-L, and FOGALS-g3 models were excluded from the 18 CMIP6 models in Table 1 in the calculation of the MME. As the data are only used as the initial conditions, we only corrected the climatological mean bias in the MME soil temperature against the ERA5 dataset using the bias correction method proposed by Holland *et al*.^17^. The MME soil moisture was corrected by multiplying a scale factor defined by the ratio of the climatological mean derived from the ERA5 dataset to that derived from the MME over the time period 1979–2014. Both the bias-corrected soil temperature and moisture data therefore have an ERA5-based mean climate.

### Evaluation methods

To comprehensively compare the performance of bias-corrected GCM data with other CMIP6 models, we computed commonly used statistics, such as the mean error, correlation coefficient, standard deviation and centred root-mean-square difference (cRMSD)^40^. To assess the overall performance of the climate model in simulating multiple fields, we also used a multivariable integrated skill score (MISS). The MISS is defined based on vector field statistics and can summarize the overall performance of the model in simulating multiple fields^41–43^. The model performance improves monotonically with an increase in the MISS. The MISS approaches 1 when the modelled multiple fields are close to the observed values. The MISS can therefore be used to rank the performance of various CMIP6 models in simulating multiple fields.

## Data Records

The dataset includes three surface variables and eight upper air variables for three sets of bias-corrected CMIP6 data, the historical data from 1979 to 2014, and SSP245 and SSP585 from 2015 to 2100 (Table 2). We provide the data at a horizontal resolution of (1.25° × 1.25°) and six-hourly intervals. The upper air variables consist of 14 pressure levels (1000, 925, 850, 700, 600, 500, 400, 300, 250, 200, 150, 100, 70 and 50 hPa). All the bias-corrected data were stored in a self-describing NetCDF format^44^. The NetCDF files are named “atm_experiment_yyyy_mm.nc4”, where “experiment” denotes the historical, SSP245 or SSP585 experiments. “yyyy” and “mm” denote the year and month of the data, respectively. Each file includes all the six-hourly data for one month of a year. The complete dataset is about 1.9 TB in size. All the data are freely available at the China Science Data Bank^45^.

**Table 2:** Bias-corrected variables.

Variables	Acronym	Vertical levels	Historical time period (1979–2014)	SSP245 scenario (2015–2100)	SSP585 scenario (2015–2100)
Sea surface temperature	tos	1	√	√	√
Sea-level pressure	psl	1	√	√	√
Surface pressure	ps	1	√	√	√
Air temperature	ta	14	√	√	√
Zonal wind	ua	14	√	√	√
Meridional wind	va	14	√	√	√
Relative humidity	hur	14	√	√	√
Geopotential height	zg	14	√	√	√
Soil moisture	tsl	4	√	√	√
Soil temperature	mrsol	4	√	√	√

## Technical Validation

The ERA5 data form one of the best reanalysis datasets with a full set of variables available for driving RCMs despite its own biases^38,46,47^. We used the ERA5 data as the “target” data based on which bias correction is carried out. The bias-corrected GCM data were compared with the ERA5 data to validate the bias correction method. The difference between the bias-corrected GCM data and the ERA5 data represents the remaining errors that are not corrected. A smaller remaining error indicates a better performance of bias correction method and vice versa. In general, a smaller remaining error suggests a better quality of bias-corrected GCM data.

### Time series

**Fig. 2:**
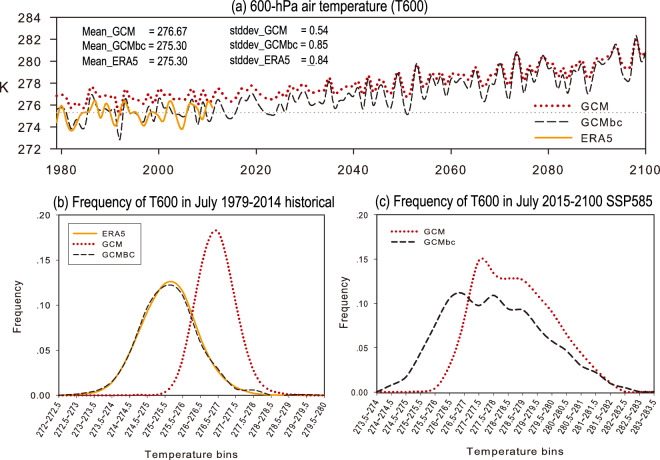
Comparison of the original and bias-corrected GCM (GCMbc) data with the ERA5 data during the historical time period (1979–2014) and the SSP585 scenarios (2015–2100). (**a**) 600-hPa air temperature (T600) in the Northwest Pacific (10°N, 135°E) at 0000 UTC on July 15 from 1979 to 2100. The 36-year averaged (1979–2014) value and temporal standard deviation for each dataset are shown in the upper-left of panel (**a**). The frequency distribution of T600 in July during the time period (**a**) 1979–2014 and (**b**) 2015–2100 under SSP585 scenarios. The frequency (percentage) is computed based on the instantaneous data at 0000 UTC in July. A total of 1116 days (31 days × 36 years) and 2666 days (31 days × 86 years) are included in the statistics for the historical and future time periods, respectively.

We used the 600-hPa air temperature (T600) in the Northwest Pacific as an example to show intuitively the changes induced by the MVT bias correction. The time series of T600 derived from the original GCM and bias-corrected GCM (GCMbc) data were compared with the ERA5 data (Fig. 2). The original GCM data overestimate the air temperature by 1.37 °C during the historical period (1979–2014) relative to the ERA5 data (Fig. 2a). The mean bias is completely removed in the bias-corrected GCM data. Compared with the ERA5 data, the original GCM data underestimate the amplitude of the interannual variability, characterized by a standard deviation of 0.54 in the GCM against 0.85 in the ERA5 data. The variance bias is also removed in the bias-corrected GCM data. In addition, the GCM and GCMbc data show different long-term trends because the GCMbc data follow the non-linear trend of the ensemble mean of the 18 CMIP6 models. Note that the MVT bias correction method proposed here affects the GCM mean, variance and non-linear trend, but does not affect the phase of the interannual variation. The GCMbc data inherit the phase of the interannual variation from the selected GCM (i.e. the MPI-ESM1-2-HR data).

The frequency distribution of the 600-hPa air temperature indicates that the GCM significantly overestimates high-temperature events, but underestimates low-temperature events relative to the ERA5 data (Fig. 2b). By contrast, the bias-corrected GCM data (i.e. GCMbc) shows a frequency distribution very similar to that of the ERA5 data. This indicates that the MVT bias correction method significantly improves the characteristics of GCM extreme events. Under the SSP585 scenario (Fig. 2c), the temperature is a non-stationary time series in association with significant global warming. Consequently, the frequency distribution of temperature does not follow a normal distribution. The change in the frequency distribution during the future period is similar to that during the historical periods and is characterized by a shift towards a colder climate and an enhanced variance.

### Climatological mean

**Fig. 3:**
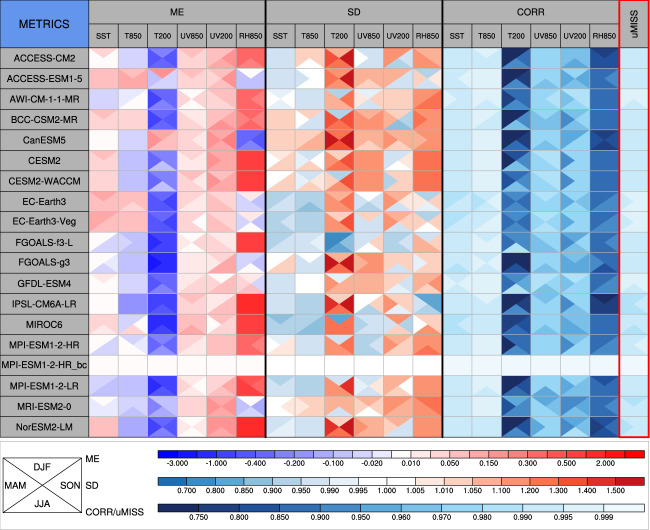
Statistical metrics used to measure the performance of the CMIP6 models in simulating the climatological mean (1979–2014) of multiple variables. SST, sea surface temperature; T850, 850-hPa air temperature; UV850, 850-hPa vector wind; RH850, 850-hPa relative humidity; T200, 200-hPa air temperature; UV200, 200-hPa vector wind in the global model. ME is the mean error, SD is the spatial standard deviation and CORR is the correlation coefficient of the climatological mean field. uMISS is the uncentred multivariable integrated skill score that summarizes the overall performance of a model in simulating multiple variables. All the statistics are normalized by dividing the observed standard deviation of the corresponding variable. MPI-ESM1-2-HR_bc is the bias-corrected GCM data. All models are evaluated against the ERA5 data. Lighter colours represent a better model performance.

The climatology of the bias-corrected GCM data and the 18 CMIP6 models were assessed against the ERA5 dataset using multiple statistics – for example, the mean error, the spatial standard deviation and the correlation coefficient for multiple variables. All the statistics were normalized by dividing the observed standard deviation of the corresponding variable to facilitate intercomparison between different variables. The results suggest that the CMIP6 models show a greater mean error in the 200-hPa air temperature and 850-hPa relative humidity (Fig. 3). Most of the CMIP6 models underestimate the 200-hPa air temperature, as indicated by negative mean errors ranging from 0.3 to 2 times the standard deviation. By contrast, 12 out of the 18 CMIP6 models significantly overestimate the 850-hPa relative humidity by about 0.3–2 observed standard deviations. The CMIP6 models also show a relatively poor modelling performance in the spatial pattern of the 200-hPa air temperature and the 850-hPa relative humidity, characterized by smaller correlation coefficient than for the other variables. For the climatological mean of multiple variables, EC-Earth3, EC-Earth3-Veg, AWI-CM-1-1-MR, GFDL-CM4 and MPI-ESM1-2-HR are ranked as the top five of the 18 CMIP6 models. The mean errors in the bias-corrected GCM data (MPI-ESM1-2-HR_bc) are completely removed. The climatology of the bias-corrected GCM is therefore the same as that of the ERA5 dataset during the historical time period.

### Extreme values

**Fig. 4:**
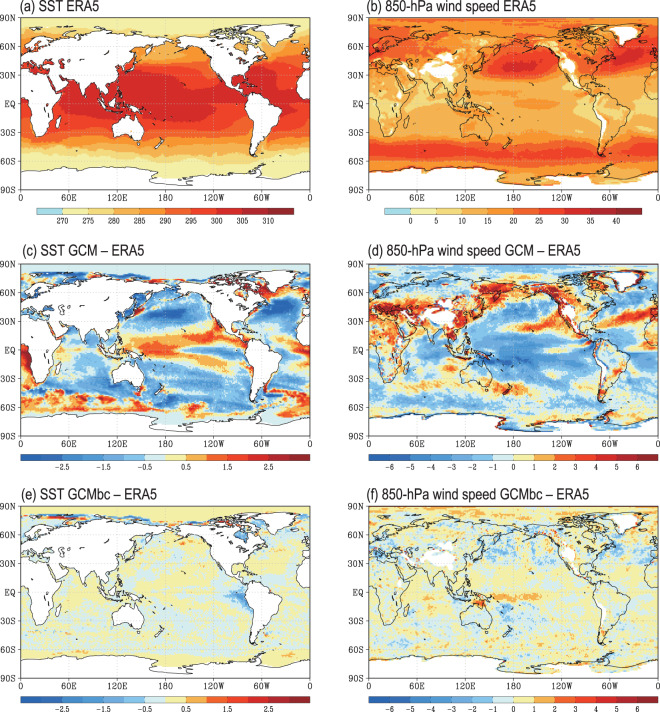
Comparison of the 95th percentile of the daily SST (°C) and the 850-hPa wind speed wind (m s^−1^) during July from 1979 to 2014 against the ERA5 data. (**a,b**) ERA5, (**c–f**) difference between the GCM (GCMbc) and the ERA5 data.

The extreme values in the large-scale forcing data will probably affect the RCM simulations through the lateral and underlying boundary conditions. To examine the impact of the MVT bias correction on extreme values, we calculated the 95th percentile of the daily SST and 850-hPa wind speed during July from 1979 to 2014 (Fig. 4). The GCM (i.e. MPI-ESM1-2-HR) overestimates the high SST extremes over the intertropical Pacific convergence zone and the South Pacific convergence zone by 1–2 °C but underestimates the high SST extremes in the central North Pacific and Atlantic oceans by about 2 °C (Fig. 4c). In the bias-corrected GCM data, the bias of the high SST extremes is generally <0.5 °C, except on the eastern coast of the central Pacific, which has a negative bias of about –1 °C (Fig. 4e).

Wind is another key variable that transfers energy and moisture into RCMs through the lateral boundary conditions. The RCM simulation is strongly affected by the wind circulation, especially when there is an inward component^48,49^. The GCM significantly overestimates the 850-hPa wind speed over East Asia, South Europe–West Asia and western North America by about 3–5 m s^–1^, but underestimates that over most parts of the Pacific Ocean (Fig. 4d). These biases are significantly reduced in the bias-corrected GCM data, with a typical wind speed bias <1 m s^–1^. We did not directly correct the biases of extreme values. However, the bias correction to the mean and variance biases will, in turn, improve the extreme values.

### Long-term non-linear trends

**Fig. 5:**
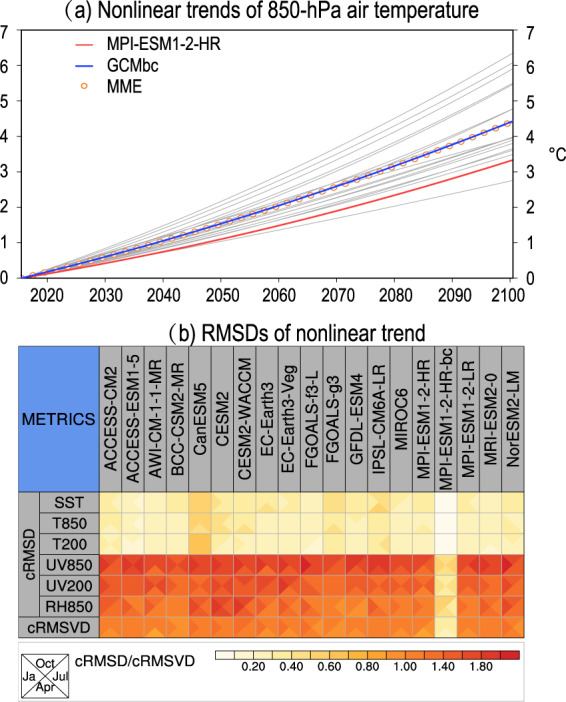
Comparison of the non-linear trend of various CMIP6 data against the MME mean in July from 2015 to 2100 under the SSP585 scenario. (a) Global 850-hPa air temperature. The orange open circles, red line and the blue line represent the MME, the MPI-ESM1-2-HR model and the bias-corrected MPI-ESM1-2-HR model, respectively. The grey lines indicate the other 17 CMIP6 models. The value of the non-linear trend in the year 2015 was subtracted from the original non-linear trend. (b) The root-mean-square differences in the non-linear trends between the individual CMIP6 models and the MME. As in Fig. 3, the cRMSDs are normalized by dividing the observed standard deviation of the corresponding variable. The cRMSVD represents the overall RMSD of all variables. The triangles in each grid cell represent the statistic in January, April, July and October, respectively.

Figure 5 shows the non-linear trends of the January-mean 850-hPa air temperature from 2015 to 2100. Under the SSP585 scenario, the CMIP6 models show a 3–6.8 °C increase in the non-linear trend of the global mean 850-hPa air temperature across 18 CMIP6 models by the end of the 21st century relative to the year of 2015. The CMIP6 models clearly still show a large uncertainty in the projection of the global mean 850-hPa air temperature. The bias-corrected GCM shows a 4.2 °C increase in the 850-hPa air temperature against the 3.5 °C increase in the MPI-ESM1-2-HR model by the end of the 21st century. Note that air temperature generally shows a clear non-linear trend^50^, which is also identified in our result (Fig. 5a). The bias-corrected GCM data preserves the non-linear trend derived from the MME. This is one of the advantages of our bias correction method relative to the previous trend-preserving bias correction methods devised using linear trend^21,22^

To more comprehensively compare the difference in the non-linear trend among the various CMIP6 models, we computed the cRMSD of the non-linear trend of each model relative to that of the MME (Fig. 5b). A smaller cRMSD indicates that the non-linear trend of the corresponding model is closer to that of the MME. Compared with all of the CMIP6 models evaluated, the bias-corrected GCM shows the minimum cRMSD for all variables. The cRMSDs are reduced by about 70–90% after bias correction. Note that the non-linear trend of the bias-corrected GCM should be the same as that of the MME for each six hours and each day of the year. However, the non-linear trends in Fig. 5b were computed using the monthly mean data rather than the six-hourly data. The anomalous time series were multiplied by different scaling factors on different days during the variance bias correction. The monthly mean of the bias-corrected data will therefore show non-linear trends slightly different from those of the MME.

### Intervariable dependency

It has been argued that the independent bias correction applied to individual variables can disturb the intervariable dependency, which may degrade the RCM simulation^28,51^. To examine the impacts of bias correction on the intervariable dependency, we computed the geostrophic wind based on the geostrophic balance:

$${u}_{g}=-\frac{1}{f}{\left(\frac{\partial {\rm{gz}}}{\partial y}\right)}_{p}$$

$${v}_{g}=-\frac{1}{f}{\left(\frac{\partial {\rm{gz}}}{\partial x}\right)}_{p}$$

where *u*_*g*_ and *v*_*g*_ are the zonal and meridional components of the geostrophic wind, respectively, *g* is the acceleration of gravity, *z* is the geopotential height and *f* is the Coriolis parameter. The geostrophic wind is a result of the balance between the horizontal pressure gradient and the Coriolis force. The ageostrophic wind (i.e. geostrophic wind deviation) is defined by the vector difference between the real wind and the geostrophic wind:

$${u}_{a}=u-{u}_{g}$$

$${v}_{a}=v-{v}_{g}$$

The ratio of the ageostrophic wind speed to the real wind speed is calculated by

$${\rm{R}}=\frac{\sqrt{{u}_{a}^{2}+{v}_{a}^{2}}}{\sqrt{{u}^{2}+{v}^{2}}}$$

R can also be interpreted as the percentage of the geostrophic wind deviation to the real wind speed. In general, R is expected to increase if the bias correction significantly disturbs the balance between the horizontal wind and the geopotential height. The difference in R between the GCM and GCMbc can therefore indicate the impact of bias correction on the dependency between the wind and the geopotential height. For comparison, we also calculated R for the ERA5 data.

**Fig. 6:**
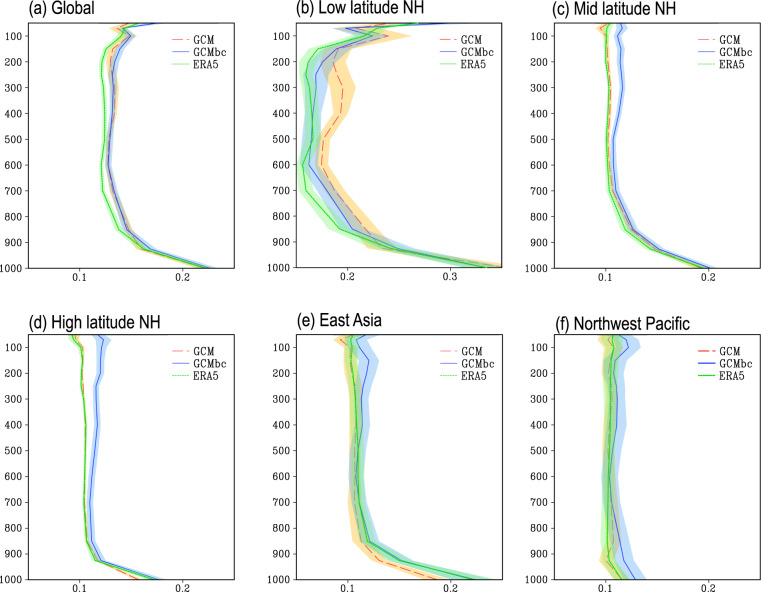
The ratio (R) of the ageostrophic wind speed to the real wind speed averaged over various regions in July 2014. The shading shows one standard deviation. The ageostrophic wind and the real wind are calculated using the six-hourly data for July 2014. The area-mean R is computed in mid (20–50°N, 0–358.75°E) and high latitudes (50–90°N, 0–358.75°E) of the northern hemisphere, East Asia (10–55°N, 105–140°E), and the Northwest Pacific (10–40°N, 135°E–180°). Grid cells with wind speeds <2 m s^–1^ were excluded from the statistics.

The vertical profile of the global mean R in the GCMbc data is very close to that in the GCM data and both show a greater R than the ERA5 dataset (Fig. 6). At low latitudes, the MVT bias correction leads to a decrease in the geostrophic deviation because the GCM biases are corrected based on the ERA5 data and the geostrophic deviation is smaller in the ERA5 dataset than in the GCM (Fig. 6b). Bias correction causes moderate, but significant increases in R (<0.02) in the mid- to high-latitude northern hemisphere. A comparison of the GCMbc data with the GCM data in East Asia indicates that bias correction also leads to significant increases in R. However the difference in R between the GCMbc and ERA5 dataset is less significant below 300 hPa (Fig. 6e). The MVT bias correction has minor impacts on the intervariable dependency in the Northwest Pacific, characterized by a less significant difference in R between the GCMbc and GCM (ERA5) datasets.

Overall, the MVT bias correction does disturb the intervariable dependency to a certain extent. The geostrophic wind deviation can increase by about 20% in the mid and high latitudes of the northern hemisphere. Globally, only 10% of the area shows significant differences in R between the GCM and GCMbc datasets. The disturbance in the intervariable dependency induced by the MVT bias correction may affect the dynamical downscaling simulation. However, the impacts are expected to be small for a long-term simulation because the imbalance only appears in the initial and lateral boundary conditions of the RCM^20^. By contrast, the bias correction-induced imbalance in the GCM dataset could significantly affect the RCM simulation if nudging was used during the integration of the RCM. In this case, we may consider reducing the strength of nudging^20^.

## Usage Notes

The China Science Data Bank provided the open access data presented in this paper^45^. The NetCDF data were compressed to save space. Users should use two attributes, scale_factor and add_offset, to unpack the variables. We provide a FORTRAN code to covert these compressed NetCDF files to WRF intermediate files^45^. In addition to FORTRAN, many different types of software are available to manipulate or display the NetCDF data (e.g. CDO, NCO, NCL, Python, Ncview and GrADS). More software can be found at https://www.unidata.ucar.edu/software/netcdf/software.html. The data provided here can be used to generate the underlying and lateral boundary conditions of the RCM.

RCMs also need the surface air temperature, soil temperature and soil moisture as initial conditions. Bias corrections of the atmospheric initial conditions are less important in long-term climate projections because the spin-up time of atmospheric variables is generally less than one month. The deep soil temperature and moisture need a much longer time to spin-up, especially in dry regions. Users can carry out an online spin-up with the bias-corrected soil temperature and soil moisture data provided in this study. Alternatively, users can generate the initial soil conditions through an offline simulation of the land surface model driven by atmospheric forcing.

## Data Availability

The code used to produce the bias-corrected global CMIP6 data is publicly available^45^. The code consists of an NCL (version 6.6.2) script to compute non-linear trends and a few CDO (version 1.7.0) scripts to regrid data and correct CMIP6 data biases.
